# Supplementary material for: MicroRNA Omics Analysis of Camellia sinesis Pollen Tubes in Response to Low-Temperature and Nitric Oxide
Source: Biomolecules. 2021 Jun 23;11(7):930. doi: 10.3390/biom11070930 (PMC8301950; doi:10.3390/biom11070930)
Supplement: Supplementary file 1 [file biomolecules-11-00930-s001.zip › biomolecules-1254255-supplementary/Supplementary Figure.pdf]

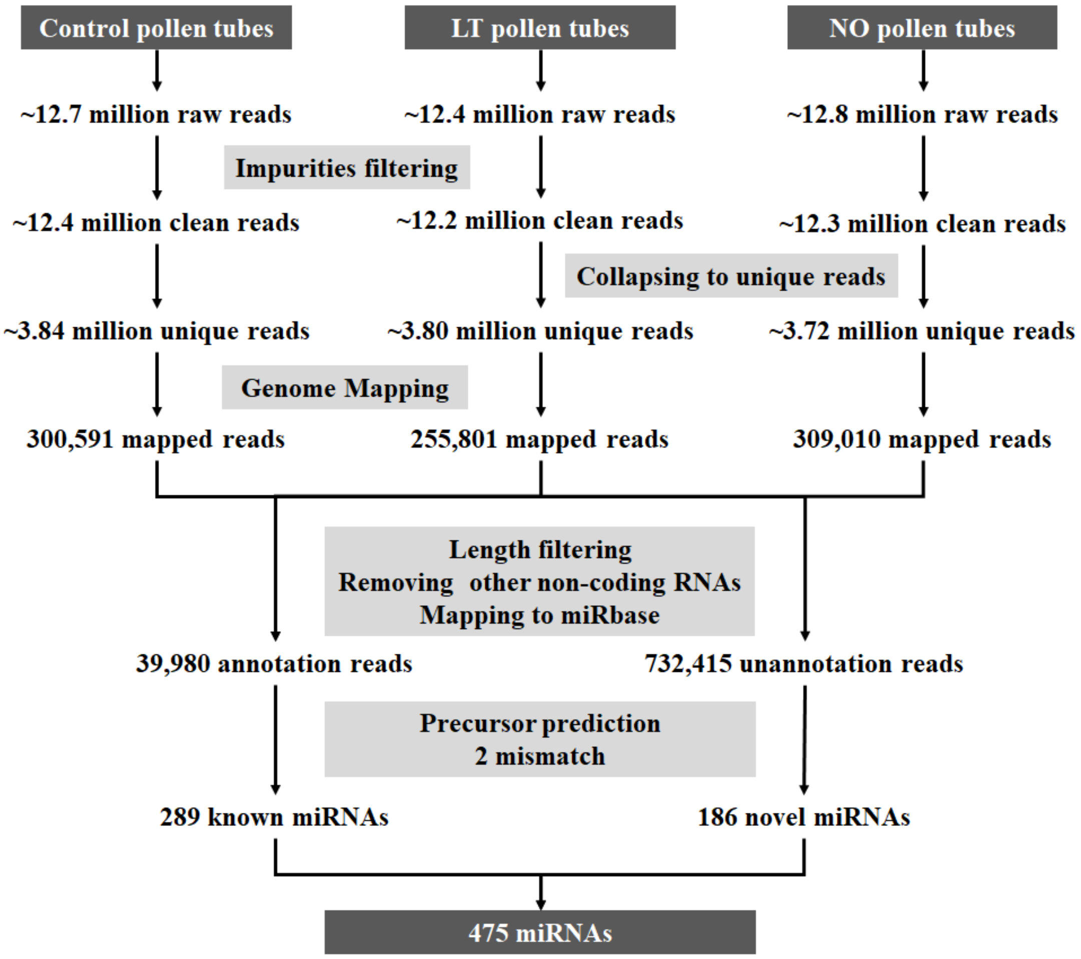

Figure S1. Workflow used for annotating miRNAs from tea pollen tubes under control, LT (low-temperature) and NO (nitric oxide) treatments.

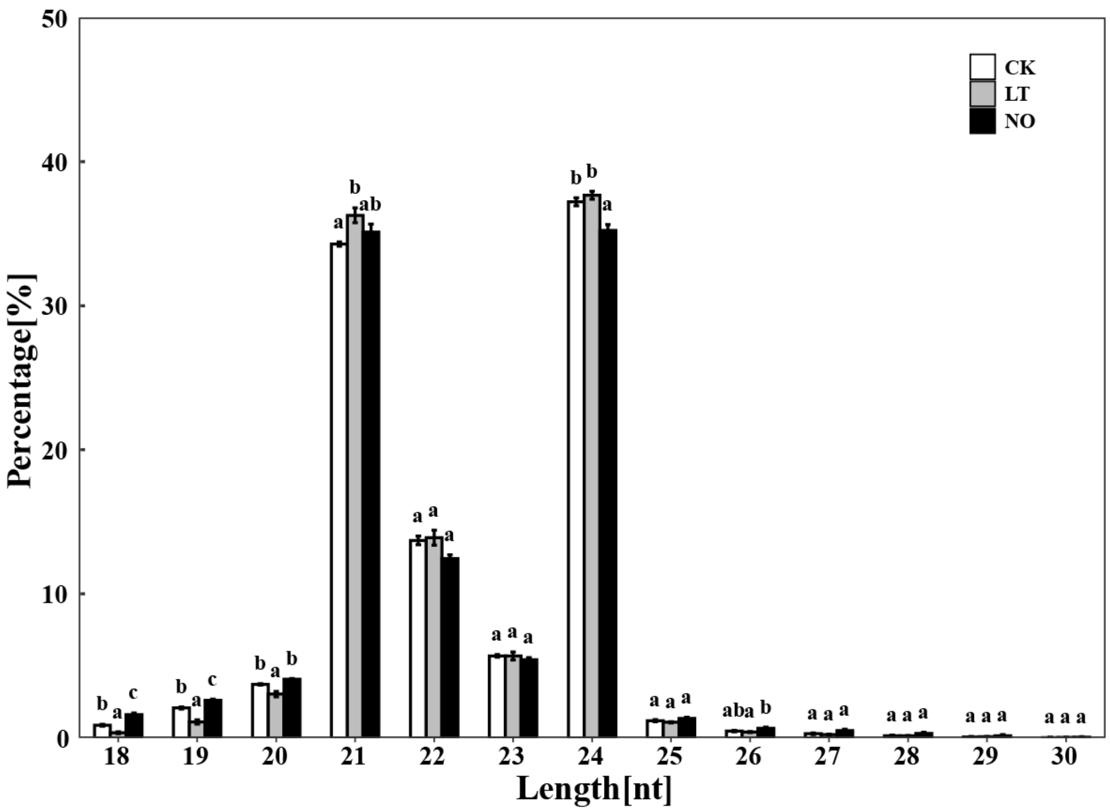

Figure S2. Size distribution for microRNAs under LT (low-temperature) and NO (nitric oxide) treatment. Different lowercase indicates significant differences among different treatments in the same length of microRNA at $P < 0.05$, as determined by Tukey HSD test.

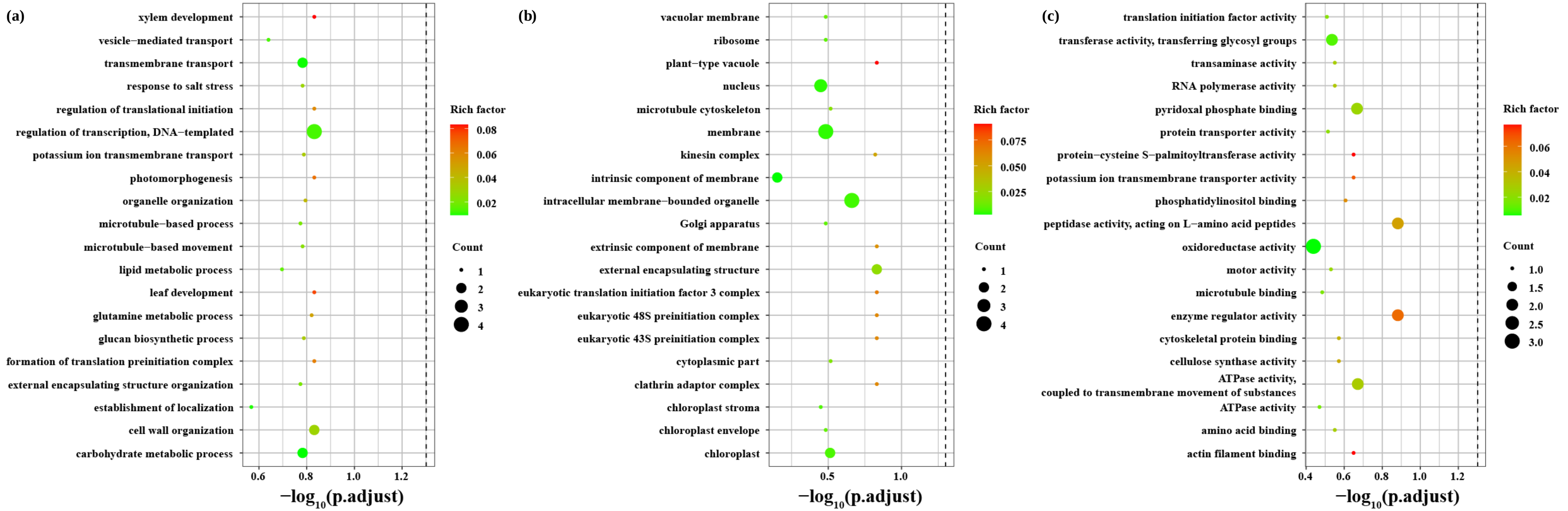

Figure S3. Enrichment analysis of gene ontology for the target genes of differentially expressed miRNAs between LT (low-temperature) and CK (control). Biological Process **(a)**; Cellular Component **(b)**; Molecular Function **(c)**. The size of bubbles represents the number of target genes enriched on the GO term. The size of Rich factor represents the enrichment degree of GO term.

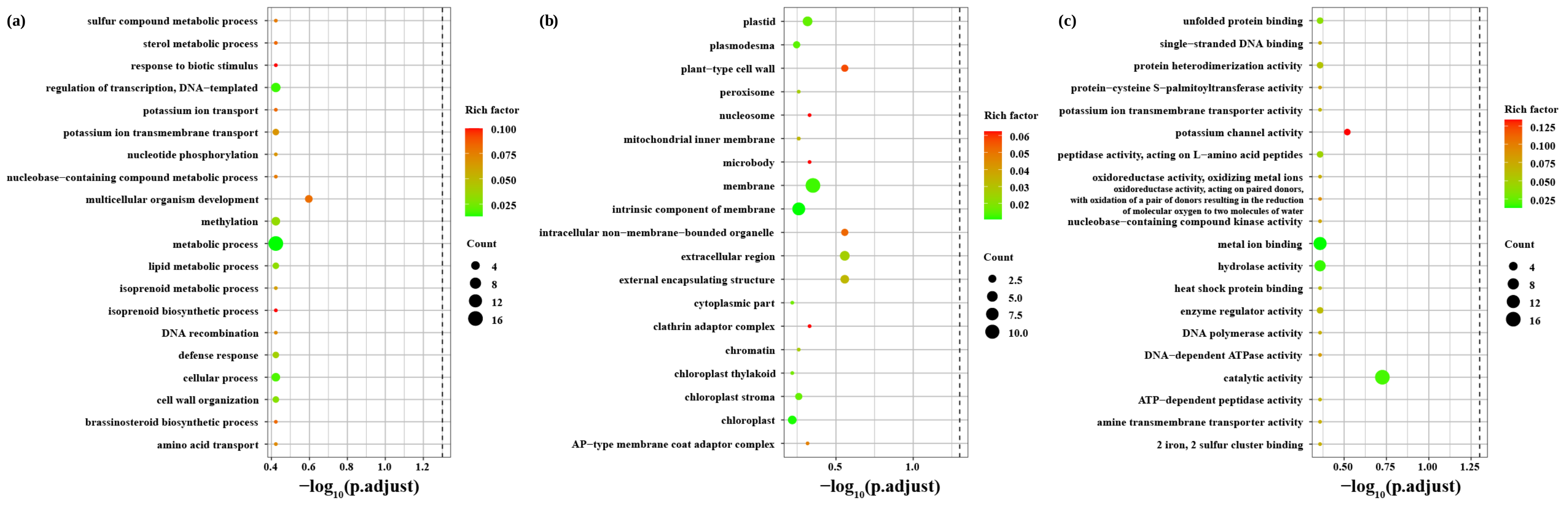

Figure S4. Enrichment analysis of gene ontology for the target genes of differentially expressed miRNAs between NO (nitric oxide) and CK (control). Biological Process **(a)**; Cellular Component **(b)**; Molecular Function **(c)**. The size of bubbles represents the number of target genes enriched on the GO term. The size of Rich factor represents the enrichment degree of GO term.

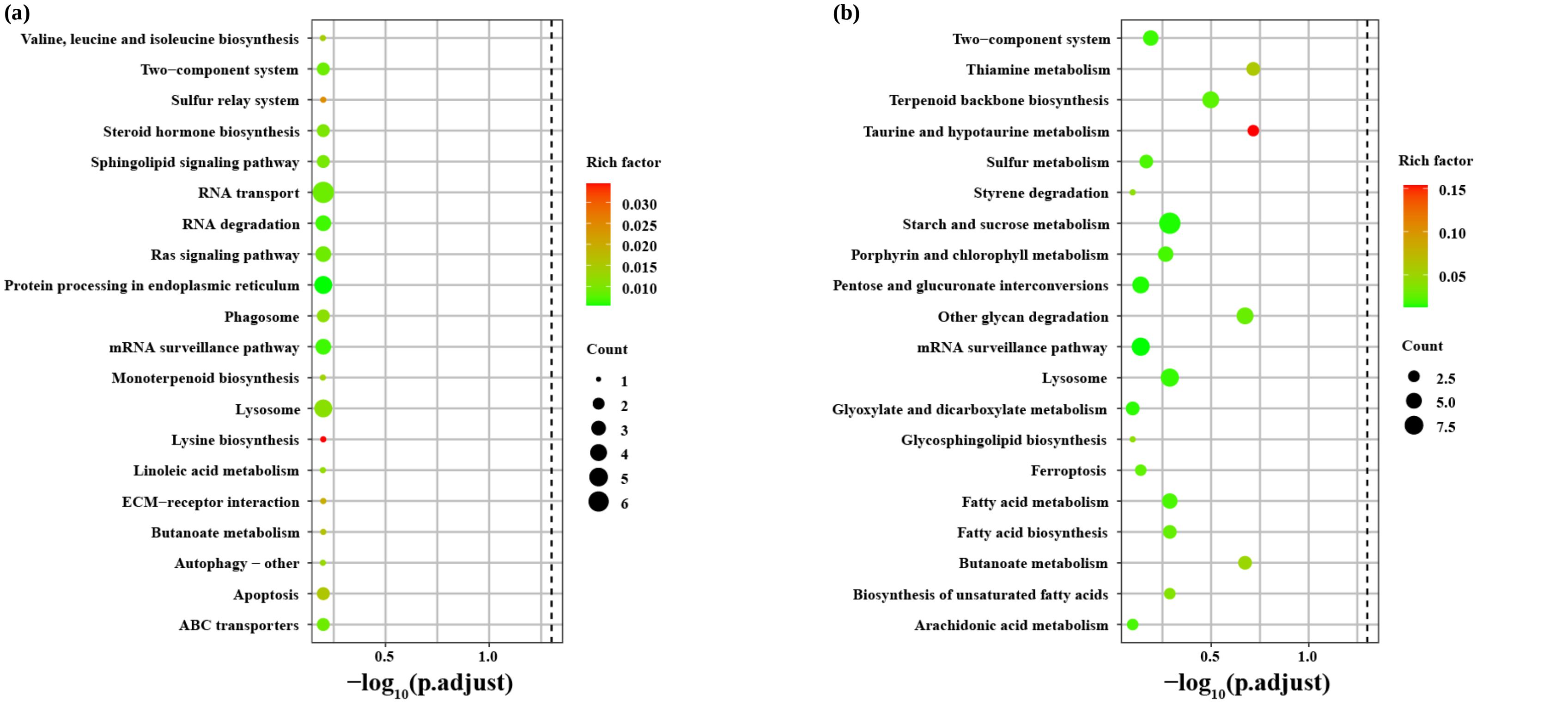

Figure S5. KEGG analysis for the target genes of differentially expressed miRNAs. between LT (low-temperature) and CK (control) **(a)**; between NO (nitric oxide) and CK **(b)**. The size of bubbles represents the number of target genes enriched on the KEGG term. The size of Rich factor represents the enrichment degree of KEGG term.
