# Supplementary material for: MicroRNA Omics Analysis of Camellia sinesis Pollen Tubes in Response to Low-Temperature and Nitric Oxide
Source: Biomolecules. 2021 Jun 23;11(7):930. doi: 10.3390/biom11070930 (PMC8301950; doi:10.3390/biom11070930)
Supplement: Supplementary file 1 [file biomolecules-11-00930-s001.zip › biomolecules-1254255-supplementary/Supplementary Table S1.pdf]

Table S1. Primer sequences information.

Name	Primer Sequences (5'–3')
miR1429-3p	GGCACGGGATTGGTATGTT
miR1535a	TGCTTGGATGGTGATGTGC
miR4416b	TTGGAGAGAGAAGACGCTATC
novel_mir_18	CTTGCAATTGTGGATTTAGGG
miR5293	TGAAGAATGAAAGGAAGCAAG
miR1873	CAATTGGTATCAGAGCTCGG
miR5380c	ATGATGATGAAGATGAACATG
miR396g-3p	GTTGAAGAAAGTGTGGACA
miR2118-5p	GTCGATGGAACAATGTAGGCA
novel_mir_37	AAGGAAGAAGAAGAAGAAG
β-actin-F	GCCATCTTTGATTGGAATGG
β-actin-R	GGTGCCACAACCTTGATCTT
TEA031966.1-F	AGCCTTTTCAAGCCCTT
TEA031966.1-R	TGTCTTTTACCATCACTCACC
TEA030352.1-F	GGAAGTATTGTGGGCTGC
TEA030352.1-R	TCCCTGTGAATGTCGGTT
TEA018965.1-F	TAAGACAAGAAACGCCTCA
TEA018965.1-R	CGTACAGTTATCCTCCACATC
TEA022010.1-F	CGCTTCGTCACCAACCTA
TEA022010.1-R	CCCACAAGATACCGCTCA
TEA000142.1-F	GTGGCTGTTTGTACGGCA
TEA000142.1-R	ATTCTTCATCTACGGACCC
TEA030640.1-F	GAACATCTGGGTTGGAATAG
TEA030640.1-R	GACATCTGAGACAGCCGAC
TEA012727.1-F	GCTATGATGATGTGGGTGG
TEA012727.1-R	GCCTCCTCAAATGCCTTC
TEA022823.1-F	CCAGTTTGGCATAAAGTAA
TEA022823.1-R	TAGAGTCACCCTAAAGAAGAT
TEA022912.1-F	AAATACGGGTCCTGGGC
TEA022912.1-R	ACCTTCAATGGTTTCACGA
TEA005168.1-F	TCCCTTCTCATCATCTCACT
TEA005168.1-R	AATACAATCTCAGCCTCTGG
